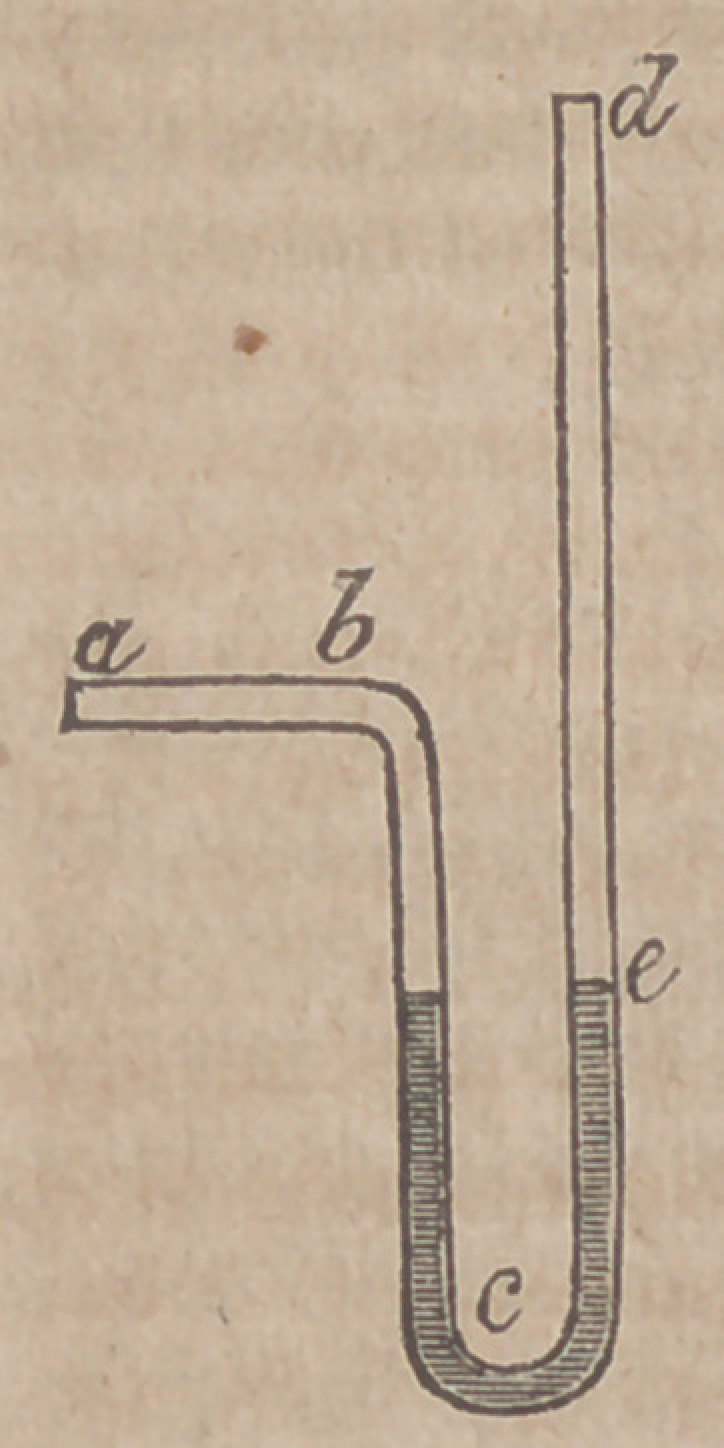# Analectic

**Published:** 1830

**Authors:** 


### MISCELLANEOUS INTELLIGENCE.

### *ANALECTIC, ANALYTICAL, AND ORIGINAL.*

ANALECTIC.

1. *Mr. Battley's Liquor Cinchonæ Cordifoliæ.*—In the first, but we hope not the last, number of Dr. Farre's Jo[illegible]nal of Morbid Anatomy, Mr. Battley has detailed some experiments which he lately made on the yellow bark. He appears to think that he has now obtained a fluid or liquor, containing *all* the medicinal principles of the bark in substance, without the inert woody fibre—consequently a preparation superior to sulphate of quinine. This is such an important subject that we shall here give the series of experiments, and the results to which Mr. Battley has come since the original experiments were published in the journal above mentioned.

"Experiments on the Cortex Cinchonæ Cordifoliæ.—Having recently been engaged in a course of experiments on yellow bark, with a view to a more correct and complete analysis of it than has yet fallen under my observation, I feel justified by the success of my labours, in submitting to the profession the following detail of my operations, for the two fold purpose of inviting their attention, generally, to the importance of pharamaceutical analysis, as subservient to the improvement of the materia medica; and particularly to the very interesting results of my present investigation into the nature and properties of bark, the actual separation of its constituent principles, and their power of combination with other substances; all with reference to the ulterior investigation of the relative medicinal virtues of the several simples and compounds, that will successively pass under our review in the different stages of this analytical process.

"1.—*Maceration.*—Ten pounds (apothecaries weight) of fine flat yellow bark, having been macerated for five hours in several gallons of distilled water, at a temperature of 150° Fahr., the infusion was of a bright yellowish red color, fragrant smell, and very bitter taste. When cooled to 110°, it became opaque, with a thick pellicle on its surface. On being exposed in the evaporating dish to a continued temperature of 150°, or thereabouts, when condensed to about a fourth of the original quantity, it began to deposit a dark tough substance, which gradually increased as evaporation proceeded, until the fluid approached the consistence of syrup, when the deposit was withdrawn, and the fluid suffered to cool to 110°. At this point it assumed the appearance, as if a quantity of milk had been diffused through it; and four or five pints of distilled water being suddenly plunged into it, the whole became disturbed; and an immediate and copious precipitation ensued, of matter in the form of masses, at first tough and waxy to the feel, but readily yielding to the pressure of the hand, and when separated from the fluid, drying very quickly, and pulverizing almost spontaneously. The fluid, after this precipitation, was beautifully bright, very fragrant, and exceedingly bitter; and, being slowly condensed, formed a semi-opaque EXTRACT,

| | lb. | oz. | dr. | gr. |
|---|---|---|---|---|
| The weight of which was | 1 | 8 | 4 | 34 |
| Ditto of deposit and precipitate together, | 0 | 4 | 0 | 30 |
| Total | 2 | 0 | 5 | 4 |

"The axtract was next diffused in distilled water, and was most easily dissolved, forming a fine rich mixture, semi-transparent, of a beautiful yellow, bitter and aromatic. Heat (130°) being applied, and *dilute* sulphuric acid added to a degree of pungency, the mixture was much brightened: after some hours it was saturated with lime. The sulphate (which was of a bright yellow) being washed and dried, was submitted to boiling alcohol; and the spirit being evaporated, the beautiful brown crystalline mass, strongly partaking both of the smell and flavor of bark. The combined matter of the deposit and precipitate was then placed in *sub-dilute* sulphuric acid. The solution was of a very deep yellow, semi-transparent, and very bitter, but at the same time pleasant to the taste. This having been decanted, and more sub-dilute sulphuric acid added, and a gentle heat applied, the second solution was in all respects similar to the first. The two solutions being then mixed together, and saturated with lime, the sulphate (of a pale reddish brown) was treated like the last, and with nearly similar effect. The residuum of the deposit and percipitate was then dissolved in successive portions of *dilute* sulphuric acid, until it ceased to impart the least bitter or bark flavour, and the several solutions being mixed together, and saturated with lime, the sulphate (which was quite white) was submitted to boiling alcohol, &c., precisely as the two former. At the last, the *insoluble* part of the deposit and precipitate being washed and dried,

| | oz. | dr. | gr. |
|---|---|---|---|
| Weighed | 1 | 5 | 54 |
| The acid solutions together having imbibed | 2 | 2 | 40 |
| Total | 4 | 0 | 34 |

This *insoluble* matter was then boiled in alcohol for twenty minutes. The spirit was both deeply tinged and flavored. The residuum was again boiled in successive portions of alcohol, until all that was soluble was taken up; when the remaining insoluble part being washed and dried,

| | oz. | dr. | gr. |
|---|---|---|---|
| Weighed | 1 | 0 | 35 |
| The spirituous solutions together having imbibed | 0 | 5 | 19 |
| Total | 1 | 5 | 54 |

"These solutions were then mixed together, and deposited (when cold) a copious percipitate, which being exposed to *sub-dilute* sulphuric acid, instantly disappeared. The acid mixture being further diluted with water, and saturated with lime, the sulphate fell instantly to the bottom, leaving suspended a gelatinous matter, which gradually subsided, and remained upon the surface of the lime in the filter. The sulphate dried of a bright yellow, and was treated like all the former. The spirituous solutions, after parting with the precipitate mentioned above, were mixed with sulphuric acid to pleasant sourness, and retained their transparency unaltered. The mixture was saturated with lime, and the sulphate (which dried of a deep red, tinged with yellow) was treated as before. The supernatant mixture was quite bright, of a deep port wine color, and astringent to the taste. Three pints of distilled water being plunged into it, the whole colouring matter was instantly thrown down, and being collected and dried weighed......1dr. 28gr.*

"II.—*Maceration.*—The whole process that has now been described, was repeated again with the residual bark. It would be tedious and unnecessary to detail the operations at length, the results being all very similar to the first, agreeing perfectly in kind, and differing only in degree, inferior (as might have been expected) both in quality and quantity to those of the first series of experiments. The only difference observable in the second instance was, that a yellow, powdery effloresence (1 dr. 28gr.) floated on the surface of the watery infusion, and that there was little or no deposit during evaporation, till after the plunge of distilled water, when the percipitation was instant and copious, as before.

| | oz. | dr. | gr. |
|---|---|---|---|
| Weight of Extract | 4 | 2 | 42 |
| Ditto Precipitate | 2 | 3 | 12 |
| Total | 6 | 5 | 54 |

"III. and IV.—*Macerations.*—The residual bark was again macerated in distilled water, a third and a fourth time, with this only difference in the present case, that the two infusions being both

"*20 grains of this imparted to sulphuric acid 2 1-2 gr.
Ditto to boiling alcohol 11gr.

extremely weak, were mixed together, and the compound operated upon in a manner precisely similar to I. and II., and with results still inferior to both in quality and quantity.

| | oz. | dr. | gr. |
|---|---|---|---|
| Weight of Extract | 2 | 5 | 4 |
| Ditto Percipitate | 0 | 7 | 0 |
| Total | 3 | 4 | 4 |

"V.—*Decoctions.*—The bark, after the four preceding macerations, was next boiled for many hours in several successive portions of distilled water. All the decoctions were mixed together, and condensed by evaporation to a state somewhat resembling mucilage in appearance, when after the plunge of distilled water, as in former experiments, percipitation ensued to the extent of—

| | oz. | dr. | gr. |
|---|---|---|---|
| | 0 | 5 | 32 |
| Extract obtained from the remaining liquid | 4 | 6 | 45 |
| Total | 5 | 4 | 17 |

"VI.—*Papin's Digester.*—The *residual bark* was then placed in distilled water in Papin's digester, and kept at a high temperature for several hours. The water was pressed off and evaporated. It had little, if any, flavor of bark, nor was the plunge of distilled water followed in this instance by any precipitation, as it was in all the former experiments. The extractive matter (wt. 3dr. 30gr.) was tough, of a dark colour, and an exceedingly disagreeable rough saltish taste. This being dissolved in cold water, formed a mucilaginous mixture of a dirty sickly brown colour, and without the least flavor of bark. Dilute sulphuric acid (to pleasant sourness) being added, the appearance remained unchanged; and the mixture being saturated with lime, and the sulphate (dirty looking) submitted to boiling alcohol, as in former experiments, there was no result of crystalline matter.

"VII.—*Acid Decoction.*—The remaining *woody fibre* having been thoroughly washed and dried, was (lastly) boiled for several hours in many gallons of distilled water, mixed with sulphuric acid, and then left to macerate for fourteen days. This last decoction was intensely bitter, and what is yet more remarkable, the fibre itself, at the last possessed so much bitterness, that it was found scarcely possible to deprive it entirely of flavor, though repeatedly deluged with water during a whole day. The decoction having been saturated with lime, the sulphate was quite white, and the supernatant liquid slightly green. The sulphate was boiled in half a gallon of alcohol, and imparted more bitterness to the spirit than any of the preceding separations of lime had done. The spirit when condensed to four ounces, was slightly tinged with brown, and strongly flavoured; and sulphate acid being added, till litmus paper indicated a trifling excess, and the mixture being set at rest, after some hours crystals began to form about the surface of the fluid, in contact with the vessel, of a much darker color, but the same aromatic odour as the fluid; and when the process of crystallization had entirely ceased, the fluid was poured off, and was still very bitter, and slightly alkaline. Having been then neutralized with the least drop of acid from the end of a glass rod, and no more crystals being seen to form, the fluid was evaporated to dryness, leaving a transparent brown substance resembling emetine, which quickly attracted moisture.

"VIII.—All the different *results* of these experiments, mentioned in order, have been arranged and deposited for the inspection of the curious, in the Museum of the Academy in London Ophthalmic Infirmary, Moorfields, where these experiments were first conducted, and where they will be again repeated, if such be the desire of the profession, for the verification of the particulars above described.

"I purposely forbear at this time to state the conclusions which I think may be fairly drawn from these experiments, or to explain more fully the particular views with which they were commenced; being impelled to this silence at present, partly, by my unwillingness to obtrude my opinions upon those, who are so well able to form their own conclusions from the facts before them; partly by my sincere wish and request to be favored with the unbiased judgments of others, in relation to all or any of the particulars that have been now presented to their notice: and lastly and above all, by my humble yet anxious hope, that the striking novelty and interest of my present communication, may have a tendency to excite encreased attention to the labours of chemical anyalysis, to awaken a spirit of inquiry favorable to future research in matters of science, respecting which, much ignorance (and its usual effect, much deception) at present prevails, and thus ultimately to lead, by necessary consequence, to important improvements both in the practice of medicine and pharmacy.

"Richard Battley.

"*Fore Street, April 24th*, 1828.

—

"[July, 1829.] P. S. Since the date of the above, I have continued my experiments on yellow bark, on a large scale and in a variety of forms; and have thus been enabled to verify the former results, and to confirm to my own satisfaction, both the principles upon which those experiments were founded, and the conclusions which I am disposed to draw from them. The issue of my investigation into this important subject, is briefly this: Analysis has failed in its attempts to discover wherein the peculiar and essential principles of bark reside; and chemistry can as yet boast no preparation of this invaluable medicine, which, combining *all* its active properties, can fairly claim to be received with confidence, as an efficient representative of this powerful agent. The boasted efficacy of the sulphate of quinine might from the very nature of the compound, have been reasonably doubted, even had success more uniformly attended its adoption in medical practice; but the repeated instances of its falure, recorded by experience, are sufficient to abate our confidence in its virtues, and to evince the want of a still more efficient preparation.

"To supply this disideratum in pharmacy, I invite the attention of the medical department to a new preparation, which I have succeeded in obtaining, by a process similar to that of the *Liquor Opii Sedativus* and which I propose to designate by the name of 'Liquor Cinchonæ Cordifoliæ,' being a concentration of all the essential properties (*with the aroma*) of bark, and proving of equal efficacy with the exhibition of bark in substance. It has been submitted to the test of experience by the highest medical authorities in London, and always with eminent success; and I think myself justified, by the numerous and decisive testimonies in its favour, in submitting it with confidence to the notice of the profession, as a valuable production of pharmaceutical art, and an important addition to the Materia Medica.

"N. B. A similar process has been tried with various other drugs, which are amongst the most useful instruments of medicine, such as Aloes, Rhubarb, Sena, Sarsa, &c. and in all cases with equal success; and specimens of all these preparations (in similar form and under similar denominations, viz, *Liquor Sarsæ*, &c.) may be seen at the Laboratory of the London Ophthalmic Infirmary, or at my private Laboratory, Fore Street, where the operations are continuous and open to the inspection of the professional public.—*Medico-chirurgical Review.*

---

2. *Peculiar Odorous Principle in the Blood of Man, distinguishing it from that of other Animals.*—Such is the title of a Memoir published in the Annals of Public Health and Legal Medicine, by M. Barruel, "chef des Travaux Cliniques," to the Faculty of Medicine. He observes, that a Medico-legal question not unfrequently arises respecting stains from blood on clothes. The chemist may pronounce that a stain is produced by blood, and not by any other coloring matter, but he cannot by possibility pretend to affirm whether that blood be or be not human. Our author had been engaged in obtaining the coloring matter from the blood, by boiling that of bulls, coagulated with a great excess of tolerably concentrated sulphuric acid. In conducting the experiments he was constantly struck with the strong smell of the stall (*odeur de bouverie*) emitted, but thought very little on the subject, till an accident occurred that drew his attention more particularly to the inquiry.

An individual, after losing considerably at play, swallowed a large quantity of opium with the view of self destruction. M. Orfila was summoned, and as a copious bleeding had been practised, resolved to ascertain whether any traces of morphine existed in the blood. M. Barruel assited, and the blood having first been coagulated in a *bain-marie*, it was triturated, without affording any peculiar odour. It was then boiled with a sufficient quantity of sulphuric acid and water, when there issued from the flask so intolerable an odour of human perspiration, that the operator was fairly obliged to make good his retreat for some seconds from the laboratory. It now struck our author that it might perhaps be possible to distinguish the human blood by this test from that of other animals, and accordingly he set to work, and ultimately arrived at the following conclusions.

1mo. The blood of each species of animal contains a principle peculiar to itself.

2do. This principle which is highly volatile, has an odour like that of the perspiration, or cutaneous and pulmonary exhalation of the animal from which the blood is obtained.

3tio. This volatile principle is combined with the blood and not to be perceived as long as the combination obtains.

4to. When the combination ceases the odorous principle of the blood is volatilized, and it becomes not only possible, but absolutely easy to recognise the animal that furnished it.

5to. In every kind of animal this odorous principle is more intense in the male than in the female; and moreover, the colour of the hair in the individual, is attended with differences in the odour.

6to. This principle is dissolved in the blood, so that it may be shewn not only in the blood, as a whole, but when deprived of fibrine, or in the serosity.

7mo. Of all the modes employed to liberate the principle in question, the sulphuric acid is the best.

In order to obtain the foregoing results, it is only necessary to pour some drops of blood, or serum into a glass, and throw on it a slight excess of concentrated sulphuric acid (about a third or half the volume of the blood) and stir it with a glass rod. The odour that arises, as was mentioned before, always resembles the sweat of the animal that supplies the blood. Detailed statements are appended respecting the odour from the blood of oxen, horses, sheep, &c. but as the above rule states the general fact, we need not particularize each animal in rotation. It became important to ascertain whether dried stains of blood upon linen would furnish any indication, and M. Barruel asserts, that provided the stain be of a certain size, it is easy to recognize the kind of blood, even after the lapse of more than fifteen days. The portion of linen stained must be cut off, placed in a glass vessel, and allowed to lie quietly in a small quantity of water for some time. When the spot is well soaked, the sulphuric acid is poured on it, and stirred with a glass rod as before. Our author is ignorant if after a greater length of time than fifteen days, satisfactory results could be obtained.

M. Barruel concludes with some hints to the Juges d'Instruction, and earnest recommendations to the profession to regale their olfactory organs. If the present become an established mode of legal diagnosis, "smelling a savory suit," will no longer be a figurative expression! Seriously, however, the facts above detailed are curious, if authentic. An experiment too to determine their accuracy would be easily made.—*Ibid.*

---

3. *Observations on the Use of the Ergot of Rye.* By Mr. Davis, Surgeon Pershore.—In a short paper published in a recent number of the Midland Reporter, we find some observations by Mr. Davis on the ergot, which we shall lay before our readers. He remarks that the ergot has strong claims to our attention, its powers being *sui generis*, and really wonderful. "Under favorable circumstances, it will bring on and end a labour in an hour, which might have gone on for days, thus saving the practitioner much harassing anxiety, as well as rescuing the most amiable and interesting portion of our species from much mental distress and acute and unnecessary suffering, and in all human probability, in many instances, from an untimely grave." But Mr. Davis is far from recommending the ergot upon all occasions. He thinks its administration requires great caution, and that it is likely to do incalculable mischief when injudiciously given.

"1. It ought rarely to be given in first cases.

"2. It ought not to be given if the pelvis be not capacious.

"3. It ought not to be given unless the parts be well dilated.

"4. It ought chiefly to be given in cases where the labor is unusually tedious, in consequence of want of action in the uterus.

"5. In many cases of hæmorrhage during parturition, and in retention of the placenta.

"If the parts be properly dilated and lubricated, and if the uterus be acting feebly and inefficiently, one dose of ergot will, I am fully persuaded, in nine cases out of ten, end the labor satisfactorily within an hour. It would be highly improper to give it in cases of impaction; in any presentation, excepting a natural one, or where there is a want of proper proportion between the child's head and the pelvis of the mother; or where there exists any rigid contraction of the soft parts.

Mr. Davis has detailed six cases where the ergot was given with great advantage. In one case, it was a first labour, and the pains were dreadfully excruciating, and recurring with scarcely any intermission. Opium was repeatedly given without effect. The os uteri was dilated, but no advance was made. A single scruple of the ergot terminated the labor in an hour and a half.—*Ibid.*

4. *Nitrate of Silver.*—The following observations on this powerful medicine were contained in a letter from Mr. Ceely, an intelligent surgeon in the country.

"In two cases of very painful digestion I used the nitrate for with months, and arrived at four grain doses. One of the patients lived on gruel two months, and subsequently small portions of fat bacon for three more; and her perseverance has gained for her that health and comfort which she had not enjoyed for five years before. In numerous other cases I have derived infinite advantage from this sedative, conjoined with the plan of diet you have so ably advocated. I have never uninteruptedly continued the use of the nitrate beyond two months; nor have I ever seen any of its ill effects on the skin. I have found this article of great use, made into a pill with extract of poppy or hemlock, or any thing similar, and introduced up the rectum in the quantity of from one to three grains, twice or thrice a day, in that simple tenesmus supervening on active or protracted diarrhœa. Its effects are more decisive and more permanent than the direct sedatives usually employed.—*Ibid.*

---

5. *Human monster with two Heads.*—This is described by Dr. De Michaelis, professor of anatomy and surgery in the Royal University of Sassari, in Sardinia, in the *Annali Universali di Medicina,* for May, 1829. Maria Teresa Parodi, at Sassari, ætat. 32, having borne eight well-formed child, was delivered on the third of March, 1829 of a female child, the upper part of which was double; it presented with the heads, which were easily protuded, the one after the other; the umbilical chord and the placenta were single. On closer examination, the child was found to be well-formed inferiorly up to the base of the thorax, which gradually widened, and at the cervical region, gave rise to four well-formed arms, and two distinct necks and heads. The following is an extract from the description.

On regarding the anterior surface of the thorax, it appears to form only one cavity, common to both children, the middle of the sternum being somewhat concave, and forming, as it were, a furrow, at the sides of which the sternal extremities of the ribs of both the children meet. In the usual situation there are two mammæ; the right of the right, and the left of the left child; and at the upper angle of the sternum, two well-formed clavicles are inserted, which belong to the external upper extremities; besides these, two smaller clavicles are seen rising from the middle portion of the sternum for the internal upper extremities, the shoulders of which are lying very near each other. The necks are quite insolated, and have the two shoulders between them; their two anterior surfaces are directed anteriorly, and toward each other, so that the children, in their natural position, embrace each other with the inner arms. The posterior surface of the thorax exhibts, in its median line, the inner ribs of both children meeting each other, and closing it posteriorly; below these, the hypochondriac regions are completely united, and inferiorly closed by a simple sacral bone, the base of which is somewhat broader than usual. Laterally from the posterior median line, the two armpits of the inner upper extremities, and the two internal mammæ are seen very near each other; the two spinal columns are slightly converged, till they meet at the sacral bone; the circumference of the abdomen does not exceed that of an ordinary infant of the same age; the navel is in the usual place; the pelvis, and the two lower extremities, exhibit nothing unusual; the external genitals are somewhat lower than usual, the labii and nymphæ are well formed, and cover three apertures, the larger of which being situated in the middle, appears to be the opening of the vagina; the two others being smaller, must, from their lateral position, be the two urethral openings; moreover, the nurse has seen the urine discharged from both openings simultaneously, from which circumstance it should appear that there is one bladder with two urethræ. The anus is single, and at the usual place.

At the first the health of both children appeared to be equally good; but, at the time of drawing up the report, nineteen days after birth, it appeared that the left was more vigorous than the right; which had an icteric hue, and was affected with slight ophthalmia. The children are suckled by the mother and a nurse; they appear to have the sensation of hunger at different times, from which it should seem that there are two stomachs; the pulsations in the præcordial regions are synchronous; the temperature of the skin, evacuation of the fæces and urine, and sleep are natural. If the genitals or anus be irritated, both children appear to feel it equally.

Since the above was in type, the intelligence of the death of this child has reached us. The head of the right side had been christened Ritta, that on the left Christina. Ritta had been ill for three days, and her illness did not appear in any degree to influence the health of Christina, so that at the moment when Ritta died, Christina was hanging on the breast of her mother and playing with her face. But suddenly she let go, heaved a sigh, and died.

The following are the most interesting details of this truly curious autopsy:—

Upon inspecting the chest, the lungs were found in a healthy state, and of a pretty regular conformation. The right lobe of the lungs of Ritta, and the left one of Christina, were evidently cramped in their development; they were consequently more contracted than their other moiety.

The pericardium was single, but enclosed two hearts so closely connected and bound together, that during life the peristaltic motions must have been simultaneous, and consequently confounded. This explains why the stethoscope transmitted but the sound of a single organ of circulation, and shows why, when life ceased in Ritta, Christina was likewise obliged to lose her's; the beating of the heart of one being locked or enchained by the immobility of the other's heart.

The organs of digestion were double, as far as the cœcum. From the cœcum, as far as the anus, there was but a single duct or passage.

There were two livers, but they united into one.—The uterus was likewise double.

The opening of the body of Christina-Ritta has not furnished any very precise idea of the nature of the disease by which death was produced. A slight adhesion of the posterior part of the pleura of the right side, with the emphysema of the lungs, indicated the existence of an inflammation of that membrane, but neither intense nor extensive, and not such as could have produced immediate death.—Death might rather, perhaps, have been imputed to a considerable accumulation of feculent matter in the rectum. Nothing could have been more easy than to have removed this accumulation, which has produced such an unfortunate result.

The remote causes of the disease by which death was produced may doubtless be traced to the delicate constitution of Ritta, and to exposure to the first colds of winter in apartments very imperfectly heated. It was with difficulty that Ritta supported the fatigue of travelling, while her sister seemed to suffer no sort of inconvenience. In the towns where they were well received, and where they could stay a long time, Ritta recovered her health with surprising rapidity, so rapidly that M. St. Hilaire says he has observed nothing equal to it in an isolated being, and ascribes it to the support received from her sister Christina, who being endowed with a very robust organization, had no doubt greatly contributed to these sudden restorations. Their stay at Lyons had been very favourable to their health. They seemed even well on their arrival in Paris, although Ritta appeared to be fatigued: but here when the severity of the season required the greatest care, the relatives, deceived in their hopes by the interference of the authorities, were reduced to a mode of life inconsistent with the care which was necessary for the preservation of their child.

It is known that Ritta only was unwell, and that Christina, whose health was good to the end, was suddenly struck dead, at the moment when her sister expired. The perfect health of Christina is the more surprising, when it is considered that the accumulation or interruption to which the death is ascribed, was situated in that part of the intestines which was common to the two sisters, but it must be remembered, that an interruption which may be slight and indifferent to a well-constituted and even vigorous body like that of Christina, might be serious to one so debilitated as that of her sister.—The heart of Ritta, compressed by that of Christina, and otherwise straightened in its movements, found itself incapable of reacting against the congestion produced by the very considerable interruption in the great intestines.

Christina-Ritta was evidently not destined to attain an advanced age. There was too much inequality between the two parts, but every thing indicates that she might have lived for several years.—Her premature death has deprived the world of many interesting observations which might have resulted from the development of two intellects existing, if not in a single organization, at least in two organizations so closely united. Of how many phenomena, psycological, physiological, and pathological, are we deprived?—The study of a being like this, arrived at an age when she could account for her ideas and sensations, would be one of the most interesting which could be offered to the meditation of the philosopher.

The possibility of the prolongation of the life of such a being to mature age appears to be demonstrated, and there is no longer any reason to doubt the general veracity of the authors who have written on such subjects. Some information has lately been given of a bicephalous girl who died in Hungary, at the age of twenty-one, the death of the two parts not being instantaneous, as in the case of Christina-Ritta, but an interval of five minutes having occurred between the death of the one and the other.—*Amer. Jour. of the Med. Sciences.*

---

6. *Experiments proving that the Nervous Tissue possesses the property of developing the Galvanic Fluid.* By Dr. Louis Beraudi, of Turin.—The numerous experiments of Wilson Philip, Edwards, Vavasseur, Aldini, Magendie, Krimer, and Wienhold, established the fact that the nervous system developes galvanic phenomena. Dr. Beraudi has instituted a series of experiments for the purpose of collecting the galvanic fluid that is thus formed, that no doubt may any longer attach to so curious a fact. The following are the results of these experiments:—

1st. Dr. B. exposed the right crural nerve of a living rabbit, the temperature of the aparment being raised to 15° Reaumur. After having carefully removed all the blood, three small and very fine steel needles were introduced into the nerve; they were separated by a small stick of sealing-wax placed horizontally. The animal evinced great pain, and at the end of a quarter of an hour it was found that the needles had not acquired the power of attracting small pieces of paper. The same needles were again introduced into the nerve, and, upon being withdrawn at the expiration of a quarter of an hour, as before, each needle was found slightly to attract small particles of rust of iron, whilst the bits of paper remained unmoved.

2d. The same experiment was repeated, on the same day, on another rabbit, but without a similar result. It had been observed that the development of the electric fluid diminished in a direct ratio to the slowness of the circulation. Pulmonary insufflation was therefore had recourse to, and at the expiration of ten minutes the magnetic property of the needles was very manifest. From this fact Dr. B. concluded that the strength of this property of the needles, produced by the nervous tissue, was in proportion to the greater or less quantity of blood exposed to the contact of the air. This remark was communicated to Professor Rolando, who recommended Dr. B. to vary the experiment, by causing the animal to respire different gases.

3d. The apartment being of the same temperature as above-mentioned, it was found that, by introducing into the lungs oxygin, hydrogen, and azote, the magnetic property developed in the nerve was very powerful after the insufflation of the first of these gases, weaker from that of the second, and not at all apparent from that of the azote.

4th. After having divided the spinal marrow of a rabbit, between the third and fourth cervical vertebræ, needles were introduced as before into the crural nerve; but in neither was the magnetic property detected, until a certain quantity of oxygen had been introduced into the lungs; it then was very manifest.

5th. The right optic nerve of a rabbit was exposed, and one needle introduced into it; which was withdrawn in eight minutes, and exhibited no magnetic property. The animal was then made to respire oxygen gas, by means of a bladder which was filled with it, but still no magnetic effect was produced. Neither hydrogen nor azotic gas produced any effect. At the expiration of an hour, the same needle was introduced into the crural nerve of the same rabbit. The animal was again made to respire oxygen gas, and a weak magnetic property was then perceptible in the needle; which was no longer developed when the experiment was repeated after having divided the spinal marrow at the part above-mentioned.

6th. The same experiment was repeated, in presence of Professor Rolando, upon the olfactory nerves, but without any result.

7th. A ligature was placed upon the crural nerve of a rabbit, into which needles were introduced *below* the ligature. No galvanic phenomena were produced. The same was also the case after the division of the nerve.

8th. Dr. B. after the manner of M. Vavasseur, endeavoured to ascertain whether this property, which was imparted by the nerve, could be communicated at any distance. To determine this fact, the crural nerve of a rabbit was laid bare and divided; and the extremities of the nerves then separated to a distance of about four lines. A needle was placed in the inferior portion of the nerve, and it was found that it had, in a minor degree indeed, acquired the magnetic property. This result confirmed Dr. B. in the opinion that the nervous influence is developed at some distance, which he had before presumed from finding the magnetic property of the needle diminished and disappear from the inspiration of hydrogen and azote.

As all philosophers believe that the galvanic fluid is capable of imparting to iron a magnetic property, and as the identity of these two fluids is admitted, the following results are deducible from the above experiments:—

1. Electricity developes itself in the nervous system.

2. The fifth and sixth experiments confirm the theory of Prof. Rolando.

3. Respiration appears to have considerable influence upon the development of the galvanic fluid in the nervous system.

4. It may be presumed that the galvanic fluid does not emanate from every part of the nervous system, but perhaps from the cerebellum, as Mr. Rolando supposes.

Lastly. That neither the olfactory nor optic nerves concur in the development of that fluid.

Dr. Beraudi does not claim the merit of first conceiving these experiments. He is aware that Beclard had before ascertained and announced, that a needle becomes magnetic from being introduced into a nerve.—*Nouvelle Bid. Med. from the Annali Univer. di Med. May*, 1829.

---

7. *Hydrophobia.*—One of the late numbers of *Graefe and Walther's Journal*, contains an account of some interesting experiments on rabid animals, by Dr. Hertwich, professor at the veterinary school at Berlin. The following are the principal results that he has obtained.

1. Of 59 dogs who were inoculated, 14 became affected with real rabies.

2. In those cases where the inoculation failed, no assignable cause of the failure could be discovered. There exists, accordingly, a peculiar disposition for the virus of rabies, as for that of other contagious diseases. (A mastiff, four years old, went through regular series of experiments without any effect being produced; while seven other dogs, who were inoculated with him, and in the same manner became rabid. Some dogs were several times inoculated before any contagion took place; in others, this effect was observed after the first experiment.)

3. It appears, accordingly, that *in cases of doubtful rabies, one or two accidental or artificial inoculations are not sufficient to serve as negative proofs of the existence of rabies.*

4. No communication of the disease ever took place by the perspiration; the contagious matter of rabies cannot, therefore, be of a volatile nature.

5. *Its vehicle is not only saliva and the mucus of the mouth, but also the blood, and the substance of the salivary glands.* It does not appear to exist in the nervous pulp.

6. The power of infecting exists at every period of the confirmed disease, and *even for about twenty-four hours after the death of the animal.*

7. The virus of rabies *appears to be inactive if administered internally:* of 22 dogs who were made to swallow it, none took the disease.

8. The application of saliva to fresh wounds appears to be as often followed by rabies, as the bites of rabid animals.

9. It is, consequently, beyond all doubt, that the disease is neither produced by the lesion, according to Girard's opinion, nor by the fear of the patient, as has been repeatedly asserted.

10. The opinion of Bader and Capello, that in dogs who had become rabid from the bite of an animal primarily affected with the disease, the saliva did not contain the contagion; and that it existed only in primary rabies, has been proved, by several experiments, to be erroneous. (This perfectly agrees with Magendie's experiments, who, having inoculated a dog with saliva of a patient affected with hydrophobia, the animal became rabid after a month, and bit two others, who were also infected, from these last, no further contagion was observed.)

11. During the period of the inactivity of the virus, there is no morbid alteration observable, either locally or in the general health of the dog thus infected, nor does the lower surface of the tongue ever exhibit vesicles. There exists, accordingly, no precursory symptoms as in other contagious diseases.

12. The disease generally breaks out within fifty days after the inoculation, or the infliction of the wound; Dr. Hertwich never observed it occur at a later period.

13. Inoculation or infection from animals affected with fierce rabies, very often produces the other modification of the disease, and *vice versa*; they are, consequently, only different forms of one and the same disease.

14. It is an erroneous opinion, that healthy dogs were able to distinguish those affected with rabies by the smell; this is not the case, nor do they abhor food mixed with the secreta or excreta of rabid dogs.—*Amer. Jour. of Med. Sci.*

---

8. *On the Effects of Camphor in a healthy individual.* By Dr. Lucas Scudery, of Messina.—It is extremely difficult from the difference of action of the same medicine upon different individuals, the age, sex, constitution, &c. of the individual influencing the effects, to arrive at any general results. The following experiments on camphor appear, however, to have been made with great care; they were performed in the presence of several persons, who themselves submitted afterwards to experiment; so that confidence may be reposed in the results.

From ten to fifteen grains of camphor taken into the stomach, produces in from fifteen to twenty minutes a decided acceleration with increased force of the pulse, which continues permanent for one or two hours. There are produced at the same time redness of the face, dryness of the skin, head-ache with vertigo, increased sensibility to light, and brightness of the eyes; injection of the conjunctiva; stricture of the chest; odour of comphor in the breath; desire to urinate; urine smelling of camphor, passed in small quantities, and producing a burning in the urethra; constipation. These symptoms generally disappear at the end of about four hours; during the night the sleep is disturbed by voluptuous reveries, with erection of the penis, and pollutions. These effects, which were produced in five successive experiments, were more decided when the camphor was taken dissolved in alcohol; their intensity was also in proportion to the increase of the quantity taken; its effects were also then more prolonged, and accompanied with feverishness. M. S. has swallowed progressively as much as two scruples of camphor at a time, in many experiments. Drs. Pasquali, Mazzetti, and Gassoni repeated these experiments, and with the same results.

A month after these experiments, Dr. Scudery wishing to ascertain what modification nitre would produce in the action of camphor, swallowed two scruples of the latter article, and five minutes afterwards he took two drachms of nitre dissolved in water. He experienced almost immediately afterwards nausea, with chilliness, and an abundant salivation. In about ten minutes the pulse lessened, but before twenty-five minutes, it had acquired new force; there supervened slight head-ache, confusion of ideas; the pain in the head gradually augmented, the light appeared stronger than natural, and objects clearer; the conjunctiva was injected; face warm; desire to urinate; pulse vibratory and frequent. In this state, Dr. S. took another solution of two drachms of nitre: immediately afterwards the nausea reappeared, but the head-ache and other phenomena disappeared; after some minutes they reappeared, but slightly. Half an hour afterwards, he passed a small quantity of urine without pain; pulse natural; during the remainder of the day no phenomena appeared, he passed a calm night; urine abundant, and depositing a sediment; he had two alvine evacuations. These experiments, like the preceding, were frequently repeated with different doses of nitre and camphor; and Mr. Mazzetti also made some trials with them.

From all these experiments, Mr. Scudery concludes—1st. That in the dose of from eight to ten grains, camphor produces in a healthy man scarcely any appreciable effect; and that in diseases it should be given to the extent of two scruples, but in divided doses. 2d. That one of the effects of camphor upon the system, is to produce an excitation, characterized by an acceleration of the circulation, and an elevation of animal heat. 3d. That it produces no irritation of the gastro-intestinal mucous membrane, that it excites neither pain nor borborygmus, but that it constipates. 4th. That it acts specially upon the genitourinary organs, augmenting the energy of their functions; voluptuous reveries, erection of the penis, sensation of heat in the urethra when urine is voided, are proofs of its stimulating action. 5th. That vertigo, the vivid impression of light on the eye, head-ache, acceleration of the circulation and excitation of the genito-urinary organs, &c. announces that camphor acts directly upon the brain and the great sympathetic. 6th. That the stimulating effect of camphor is augmented by uniting it with another stimulant, as alcohol; whilst nitre, on the contrary, diminishes its stimulating properties.—*Arch. Gen. from the Annali Universali di Med. Milan, June* 1829.

---

9. *Treatment of persons poisoned by Opium.*—M. Orfila recommends when persons have been poisoned with opium, and this poison has not been absorbed or ejected by vomiting, that the patient should be made to drink, before an emetic is administered, a strong decoction of nut-galls, which substance decomposes the opium.—*Nouvelle Biblioth. Med. July*, 1829.

---

10. *Treatment of persons poisoned with Hydrocyanic Acid.*—The researches of M. Orfila has led him to recommend the following treatment of persons poisoned by hydrocyanic acid:—1st. To give an emetic if the poison is still in the stomach. 2d. To make the patient inhale ammonia or better chlorine; to combat the cerebral symptoms by bleeding, and leeches applied behind the ears. 3d. To employ cold affusions, which are very useful. M. O. says that unless the dose of the poison taken is very large, these remedies will be successful.—*Ibid.*

---

11. *Mode of stopping Hæmorrhage by twisting the mouths of the vessels.*—M. Amussat communicated to the Royal Academy of Medicine, at their meeting of the 16th of July last, this method of arresting hæmorrhage, which was, however, not unknown to the older surgeons, and is at present practised in veterinary surgery.—Though we cannot for a moment believe that this mode will ever supersede the use of the ligature as proposed by M. A. we nevertheless think the views of M. A. of sufficient interest to lay them before our readers.

Reflecting on the well known fact that lacerated wounds are frequently not followed by hæmorrhage, M. Amussat conceived the idea, that methodically twisting arteries might perhaps be attended with the same result. A number of experiments were accordingly made on dogs, rabbits, horses, &c. and the following proceeding found to answer the purpose. An artery being cut across, the extremity is seized by means of a pair of forceps, the branches of which are closed by a spring. The end of the vessel is dragged out from the surface, for the distance of five or six lines, disengaged from the neighbouring parts, then seized with the thumb and forefinger of the left hand, and twisted five or six times upon its axis by means of the forceps in the right. The twisting ought to be continued till the portion of the vessel in the bite of the instrument is broken. The end of the vessel now forms a cul-de-sac, which resists the impulse of the blood from behind, and is seen and felt to pulsate strongly.—The twisting appears to act by rupturing the middle and internal coats, which instantly retire into the interior of the vessel, and form a sort of plug or valve, whilst the vessel itself looks like a stump, enveloped and capped by the cellular membrane. If the vessel before being twisted is not seized with the left hand, it is injured up to the next collateral vessel. M. Amussat has tried the method twice on the human subject, once after extirpation of the testicle, and once after amputation, with success. Such is the substance of the first communication to the academy; in the second, some points are touched on more at large.

In the first place, the effects of torsion or twisting are the same on arteries and veins: secondly, simple torsion, when employed to prevent hæmorrhage from arteries of considerable caliber, should consist of ten half turns; thirdly, that twenty half turns completely rupture the artery; fourthly, that after the complete or incomplete section of an artery, the torsion should be made on each extremity; fifthly, that after a twisting methodically made we need never fear secondly hæmorrhage; sixthly, that torsion employed on the arteries of the dead human subject is attended with exactly the same effects, as to the vessel, as in living animals, namely, the rupture of the inner coats and their retraction within the arterial tube, whilst the cellular membrane round then forms a kind of *capachon;* seventhly, that if the arteries are ossified, torsion breaks them; eighthly, that if we inject liquid into the twisted artery with whatever force we please, it does not escape by the twisted extremity, though sometimes the retracted inner tunics are driven out by the fluid, and the cellular membrane more or less distended; ninthly, that torsion has *all* the advantages of the ligature, *without* its inconveniences, and should instantly be adopted by army surgeons in particular.—*Journal des Progres, Vol. XVI. & Journ. Hebdom. Nos.* 43–44.—*Am. Jour. Med. Sci.*

---

12. *New mode of employing the Nitrate of Silver as a test for Arsenic.* By Patric Forbes, Professor of Chemistry, King's College, Aberdeen.—"The following mode of procedure," Dr. Forbes says, "avoids all the sources of ambiguity arising from the presence of muriatic acid, or any of its combinations, or of the alkaline phosphates; and it is so simple, that any person, at all capable of performing chemical experiments, may conduct it in a satisfactory manner.

"1. The suspected liquid must be carefully filtered, and if very viscid, may be diluted with *warm* distilled water. If after filtration it has colour, it should be diluted with distilled water till the colour has *just* disappeared. A solution of nitrate of silver, (which precipitates free muriatic acid and all its combinations, decomposes all the alkaline phosphates, and precipitates the phosphoric acid in combination with the silver, but has no effect by simple affinity on arsenious acid,) is then to be dropped into it till all precipitation ceases. The liquor must again be filtered, and solution of nitrate of silver added, when no precipitate will appear if sufficiency of nitrate of silver has been formerly used. If any precipitate do appear, an *excess* of nitrate of silver must be added, and the liquor again filtered. Take now a small glass rod, and dip the point of it in a solution of pure ammonia, and just touch the surface of the liquor with it; in a few minutes, if any arsenious acid be present, abundance of the well-marked yellow precipitate of arsenite of silver will diffuse itself down through the liquor.

"2. Great care must be taken to add very little of the pure ammonia. The reason is, that the pure ammonia in excess has the power of dissolving the arsenite of silver. This property of ammonia affords, therefore, a second decisive test; for, after the precipitate has been fully formed, it is only necessary to add ammonia in excess, when, if the precipitate be arsenite of silver, it will on stirring be perfectly dissolved, and the liquor will appear colourless as before.

"3. By adding nitric acid to this colourless liquid till the ammonia is neutralized, the precipitate of the arsenite of silver may be again made to appear, which affords a third proof of the nature of the precipitate.

"4. The precipitate may now be collected, mixed with black flux, (both being made quite dry,) put into a glass tube, and the arsenic sublimed and condensed in its metallic state.

"It will be observed, that I have not directed the contents of the stomach to be boiled before the test is applied. The reason of this is, that it is wished to prevent the union of the arsenious acid with any free alkali which may happen to be accidentally present, which would prevent the success of this process. The application of *warm* distilled water on the contents of the stomach in the filter will, however, be found sufficient to carry along with it all the arsenious acid, and will not in any way interfere with the future steps of the analysis.

"The use of nitrate of silver employed in the above manner in the detection of arsenious acid recommends itself by its simplicity, and by the ease with which the substances employed may at all times be procured. But it by no means supersedes the employment of sulphuretted hydrogen, which also affords excellent indications of the presence of this poison. Every liquor supposed to contain arsenious acid should be subjected to both these reagents; and if both these tests exhibit the appearances which ought to follow their use if arsenic be present, there cannot remain the smallest doubt on the mind of the analyst, and he will be enabled to give his testimony with the most unhesitating confidence.

"If any one wishes to satisfy himself of the accuracy of the method now detailed, he has only to make a mixture of any muriates, alkaline phosphates, solution of arsenious acid, decoction of onions, coffee, &c. and to proceed as above directed.

"It deserves attention that the precipitate must be guarded from the *direct rays* of the sun, which will immediately turn it black from the presence of nitrate of silver and vegetable matter."—*Edin. Med. and Surg. Jour. Oct.* 1829.—*Ibid.*

---

13. *Power of Sulphate of Quinine in accelerating Mercurial Action.*—Dr. Harty of Dublin has inserted in the Edinburgh Medical and Surgical Journal for October 1829, a letter, in which he offers a few remarks on the power of sulphate of quinine in accelerating mercurial action. The first cases in which he perceived this effect occurred among the female convicts in Richmond Bridewell. The disease was ague. He administered to the patients the sulphate of quinine, "directing at the same time the occasional use of the "*house pill*" consisting of calomel and scammony. Though I had seen sudden ptyalism excited by a few doses of these pills in a few of the female prisoners, I was yet much surprised at the rapidity and severity with which, on this occasion, almost all those were so assailed who were taking the quinine." Dr. Harty witnessed the same effect in the male prisoners of Newgate. In some of these he found that the quinine re-excited the mercurial action after it had apparently ceased. He details the two following cases in exemplification of these facts. *Case* 1. "A boy four or five years of age, previously very healthy, was seized with severe tertian. The tongue being foul and the belly full, directed three grains of calomel, which purged him well and relieved him much. I then administered the quinine in solution, and in about six days I repeated the calomel with similar good effect, still continuing the quinine. The ague speedily ceased, but at the expense of the worst ptyalism with ulcerated and everted gums I had seen for a long time. He did not entirely recover from its effects at the end of six weeks." *Case* 2. "A medical friend to whom I had communicated these facts, having been seized with quotidian, employed the bark liberally, and then the sulphate, without any previous use of mercury, which he dreaded from the readiness with which it usually affected his gums. The quotidian accessions continuing, though with abated violence, and the feces appearing of a highly bilious character, he was induced to take five grains of Plummer's pill (containing one of calomel) with the intention of repeating it daily. In less than six hours after the first dose, he distinctly felt the mercurial action establishing itself in the gums, and could scarcely credit his feelings until the facts already detailed were recalled to his recollection."—*North Am. Med. and Surg Journal.*

---

14. *Tetanus cured by the Acetate of Morphia, applied externally.* Omodei's Annals for May 1829, contains the details of a case of tetanus, in which Dr. G. Cerioli of Cremone, resorted with the fullest success to the acetate of morphia applied to the denuded skin. It does not appear that the disease was completely developed, as the abdominal muscles are only said to have been spasmodically contracted, and those of the extremities and body only momentarily so. Trismus was complete, as it was with the utmost difficulty that the mouth could be opened. After using without success a variety of means, as blisters to the wound, cataplasms, opiate and camphor liniments, acetate of morphia administered internally, bloodletting, leeches, sudorifics, &c. Dr. Cerioli directed a blister to be applied to the back of the neck, and a quarter of a grain of the acetate of morphia to be sprinkled on the denuded skin. The application was commenced on the first of November, about ten days affer the appearance of the tetanic symptoms. On the 10th of November the patient was so much better, that the remedy (the dose of which had been increased a few days before) was discontinued. On the 16th the patient was discharged cured.

While on the subject of tetanus we must mention that at a meeting of the Royal Academy of Medicine, Mr. Lisfranc presented a man whom he had cured of the traumatic form of the disease. In this case emprosthotonos was completely formed, and in a few days tetanus became general. The treatment, which lasted nineteen days, consisted in the following means: 1. Eight copious venesections.—2. Seven hundred and ninety-two leeches (equivalent to upwards of two thousand of our own) applied along the spine, with the exception of fifty, which were directed to the epigastrium, with a view of removing gastric irritation manifested at one period of the disease. 3. Several full baths. 4. Laudanum injections, twice a day.

On the twenty-fourth day after the appearance of the disease, the patient was pronounced cured, and a few days after took a walk in the court of the hospital. Notwithstanding the copious abstraction of blood to which the patient was subjected, the pulse retained its strength, and no signs of debility were noticed. We are indebted for this article to the Journal des Progres, No. 16.—*Ibid.*

---

15. *Dr. Wedemeyer on the Circulation.*—Dr. Wedemeyer published a book at Hanover, in 1828, on the subject of the circulation. The following are some of the results obtained by him.

1. In the natural state of pulsation of the arteries, the sort of contraction obtained by Dr. Hastings, by the application of stimulants, and noticed by John Hunter and some other physiologists, can never be observed.

2. Bichat and Parry are right when they assert that at each pulsation there is always a slight augmentation of the calibre of the artery; but this increase is so slight that it can be with great difficulty appreciated.

3. All the phenomena of the arterial pulse may be imitated by injecting with a syringe, successive jets of water into the arteries, even several hours after death.

4. The pulse is caused entirely by the impulsion communicated by he heart to the blood; an impulsion by which the artery is partly dilated and partly moved from its place.

5. All the phenomena of the arterial circulation may be referred to the elasticity of the coats of this order of vessels.

6. Lastly, the great arteries contribute to the movement of the blood only in this, that they re-establish, or return by means of their elastic coats, the force employed in dilating them.—*Archives Aug.*

In the Archives for September there is a continuation of the notice of Dr. Wedemeyer's work. Dr. Wedemeyer, says the writer in the Archive, seems inclined to adopt the singular opinion, emitted by Gruithuisen, Dollinger, and Oesterreicher, on the origin of the blood-globules, which is, that they are, in great measure, formed of globules detached from the parenchyma of the various organs. These *savans* maintain that the globules may not only be seen passing from the *last capillaries* into the substance even of the tissues in which the vessels are distributed, but also that the globules, which for a certain time had constituted part of any tissue, may be seen returning to the general circulation by entering the nearest capillary vessel: whence they conclude that the blood is elaborated in the proper parenchyma of the organs. Dr. Wedemeyer, admitting that the chyle contains globules, and that it is the manifest source of nutrition and growth, cannot deny that it must play a principal part in the hæmatosis—yet remarks that, nevertheless, the globules are not immediately formed there, but only after having undergone some preliminary metamorphosis in the substance of the tissues.

For our own part, we have always supposed that if globules enter as a component or constituent part into the chylous fluid, and do not find their way into it by accidental absorption or transfusion, the absorbent vessels must be regarded as possessed of the power of communicating, if not the first, almost the very first vital properties to dead matter. A man may dine on a boiled potato or a roasted turkey, both of which specimens of matter are doubtless as completely divested of life as any stone or clod; and in less than two hours a good part of the potato or turkey shall be found restored to the enjoyment of vital properties as schyle globules, to be soon rendered more vital as blood globules, and perhaps to attain to an additional grade by being deposited as component part of a tissue; now the change from the state of boiled potato to that of retina, or sympathetic nerve, or medulla oblongata, like a succession of *avatars*, leads only to perfection, and if we hold to our solidism, we shall continue to believe that the state of *deposit* as tissue is the highest vital state to which matter can attain; consequently, the blood globules, being less vital, less perfected, are not identical with the globules of the ultimate fibre; we have reason therefore to believe that the blood globules are not constituted out of the detritus of our organs, or their effete portion.

In regard to the structure of the minute vessels, Dr. Wedemeyer says that the white elastic coat, so visible in the large arteries, accompanies the small branches, and is discoverable in tubes whose calibre is only one-sixth of a line; but in certain states of minuteness, both the elastic and lining coats disappear entirely, and the small vessels are then only so many canals, formed in the substance of the tissue, and wholly without any proper membrane, or membrane of their own; their coats consisting simply and solely in the tissues in which they are distributed. This opinion he attempts to fortify in the following manner: 1. It is impossible to discover, even with the microscope, any membrane interposed between the parenchyma of the tissue and the blood that moves in its capillary vessels. To this it might be objected, that the vessels are perfectly transparent when reduced to so small a size, (even the large veins are translucent) and consequently they cannot be seen, while the opaque blood in them is clearly seen. The pericardium is said to be so transparent that the heart is visible as clearly through it as the iris through the cornea.—2. The facility with which certain globules are seen to detach themselves from the current, or mass in which they were moving, and go to mingle with the globules which constitute the adjacent tissues.—This we cannot adopt as proof of a parenchimal circulation, inasmuch as we have often with the microscope seen that a vessel capable of admitting only one globule into its calibre, has suddenly become enlarged so as to admit two or more in rank; and also, a vessel freely admitting a stream of globules, in what may be called single file, has become suddenly so contracted as to deny entrance to them entirely; it is this liability to alternate enlargement and diminution that has probably deceived both Dr. Wedemeyer and his supporters. 3. The rapidity with which the blood opens to itself new passages in the midst of the tissues. 4. The impossibility of conceivtng how nutrition and absorption should be effected through the parietes of vessels. Dr. WEDEMEYER, in our opinion, removes no difficulty by supposing a parenchymal circulation. When blood or serum is extravasated, we know very well that the result is an acchymosis or anasarca, which is slowly removed by a long process of absorptions: and yet the gentleman leaves ten million capillary vessels opened in the midst of all the tissues, to pour out their fluid ad libitum. The thought is one that we cannot entertain for a moment.

It is best to adhere to the ancient doctrine, that extravasated blood is blood escaped from its vessels; if Dr. WEDEMEYER's opinion be correct, we cannot conceive how the blood in a blushing cheek, which ought to be considered as extravasated, could so soon give place to, or succeed to a deadly paleness. Let us not *finesse* too much in anatomy, it is enough to play *ad libitum* in our pathology and practice. We should be glad to retain one part of our science in the domain of those that are denominated exact.—*Ibid.*

---

16. *Treatment of Gonorrhœal Ophalmia.*—The *Clinique*, for August 22d 1829, contains an account of the mode of treatment employed by Dr. EDWARD EISSEN against gonorrhœal opthalmia.—Not having that journal before us at this moment, we make use of an extract from the article in question, published in the September number of the *Archives Generales.* Dr. DUPUYTREN, it must be premised, treats this kind of opthalmia by venesection, leeches to the inferior palpebra, and next by insufflations of calomel, and the application of laudanum to the eye. Dr. EISSEN regards the second part of Dr. DUPUYTREN's treatment as perturbative and dangerous, and attributes to it the ulceration of the cornea and the coagulation of the humours situated between the laminæ of this membrane.—The method employed by BEER, RUST, ASTLEY COOPER, AND HESSERT, appears to him far preferable. It consists in combating the inflammation by energetic antiphlogistic means. Bleeding is to be pushed very far, but leeches to the lid must be avoided, on account of the irritation produced by the bite of the animal. Cups to the temples may be advantageously substituted. As soon as the first indication is fulfilled, a revulsive action is to be obtained by blisters to the arms or back of the neck, and particularly by the use of calomel given in large doses, and in combination with some active purgative, so as to guard against a salivation. To obtain a full effect from this revulsion on the alimentary canal, it is necessary to continue it until it produces an artificial disease, against which the organic reaction is necessarily called, which alone can occasion a sufficient reaction. This artificial affection is announced by borborygmæ, by green and fetid stools, by a pallid countenance, pinching of the alæ nasi, coldness of the extremities, and a metallic taste in the mouth, which the patient complains that he experiences."—The only local application to be employed consists in tepid mucilaginous decoction. Dr. EISSEN adds that astringent collyria are only admissible when the inflammation has completely disappeared, and it is simply necessary to produce the contraction of the distended vessels of the conjunctiva.

After noticing this proscription of opiate stimulants and astringents in the gonorrheal form of conjunctivitis, our readers will be amused by glancing over the following article, in which will be found recommended for another form of the same disease, a widely different plan of treatment. We derive from these two articles one of two lessons: either the two forms of opthalmia are so dissimilar in nature as to require an opposite treatment, or the same form of disease may get well under very dissimilar remedies.—*Ibid.*

17. *Treatment of Opthalmia Neonatorum.*—Dr. Wishart, surgeon to the king in Scotland, has published in the October number of the Edinburgh Medical and Surgical Journal, some observations on the purulent opthalmia of infants. We shall here present a few words on the mode of treatment he recommends, premising, however, that he considers the disease as in almost every case connected with a leucorrhœal discharge in the mother. The author remarks that the following points of practice are to be attended to. "To remove if possible the leucorrhœa in the mother previous to delivery, and if this cannot be effected, to remove artificially as much of the discharge as possible from the vagina at the time of delivery, and to pay particular attention to the eyes of the child, by washing them carefully immediately after it is born." The purulent opthalmia he thinks has a fixed course which cannot be interrupted without injury to the patient. If the patient comes under his care in one or two days from its commencement, he immediately washes away the puriform matter which glues down the eyelids with a little water, and then with a small conical pointed ivory syringe injects the following eye water, at first diluted with a little warm water.

R. Sulph. Zinci. ℈i.

Aquafontis, ℥x.

Solve et edde.

Liquoris sub acetatis Plumbi. ʒss.

Tinct. Camphoræ, ʒi. or ʒii.

Misce et cola.

This is repeated three times a day in ordinary cases, or every hour or two if the quantity of the discharge is very great and is rapidly collected. If the child continues to cry more than ten minutes, the solution must be diluted. The discharge must be carefully washed away as soon as it is observed by a little of the eye water, and at night a little of the ointment of the oxide of zinc is insinuated between the eyelids. "In some cases, I have seen considerable benefit from the application of a single leech to the outer angle of the eye, especially when we suspect the inflammation is extending to the eye ball." In a few days the swelling and redness of the eyelids diminish, and the ointment may be applied after using the eye water. If the lids are very turgid they must be freely scarified, and the bleeding encouraged by fomentations. When the swelling of the eyelid subsides, and the discharge acquires a watery appearance, and the child opens its eyes better, the collyrium may be changed to a weak solution of the muriate of mercury with the addition of the vinous tincture of opium. The red oxide of mercury ointment may be substituted to the other ointment, and applied three times a day. At the end of a month the use of the syringe may be discontinued, and the collyrium merely dropped in the hollow at the inner angle of the eye, whence it can be easily made to pass under the lid.

Considerable benefit will be derived from fomenting the eyelids, for ten minutes three times a day, and at night with the same collyrium. The cloths must not be allowed to become cold.—*Ibid.*

---

18. *Iodine in Scrofula.*—At a meeting of the Academy of Sciences of Paris, held on the 17th of August 1829, Mr. Dumeril, in his name and that of Messrs. Magendie and Serris, read a report on a memoir presented to that body by Dr. Lugol, one the physicians of the hospital Saint Louis, on the use of iodine in scrofulous diseases. We have only room to state, that from the 10th of August 1827 to the 31st of December 1828, Dr. Lugol submitted one hundred and nine patients affected with the various forms of scrofula to the use of iodine. On the last date mentioned of the whole number, thirty-nine were still under treatment, they had left the hospital much relieved, four were not in the least benefitted by the remedy, and finally thirty-six had left the establishment completely cured. Among the cases treated and cured were several of a very bad kind. Dr. Lugol made use of a solution of iodine in water, in the proportion of one-half or two-thirds of a grain, or one grain in a pound of water, with the addition of twelve grains of common salt. The whole to be taken in one day. He commenced with the first, and generally prescribed the second quantity specified after using the other two months. Besides the internal use of iodine, he employed the remedy externally in the form of wash or ointment.—*Ibid.*

---

19. *On the Action of the Arteries in the Arterial Circulation.* By M. Poiseuille.—By a series of well-devised, and apparently accurate experiments, M. P. has arrived at the unexpected result, that the force of the blood in the arteries will support a column of mercury of the same height with whatever part of the course of the arterial circulation the column is placed in connexion—whether for example it is connected with the origin of the carotid, or with a branch derived by repeated subdivision from the crural artery. And consequently he concludes that the force with which a molecule of blood moves is the same throughout the whole arterial circulation. Following out these researches, he proceeds to inquire in what manner the original impulse communicated by the heart, is transmitted unimpaired to distant parts of the circulation, notwithstanding the retarding tendency of friction, and the yielding of the parietes of the vessels.

His first object here was to ascertain whether the arteries are dilated by the stroke of the heart, and impulse communicated to the blood, and what the amount of the dilatation may be. It is well known that by most physiologists a very extravagant idea used to be entertained of the amount of their dilatation; on the other hand, that the later researches of Parry, and other experimentalists have assigned exceedingly narrow limits to it; nay, that by one eminent physiologist, Bichat, it has been denied altogether. M. Poiseuille has determined the point by means of a very satisfactory set of experiments with an aparatus of his own invention, and has ascertained that dilatation is effected; but that it is so small as certainly to be indistinguishable in an artery by the unaided senses. This apparatus cannot be thoroughly described without a diagram; it will be sufficient, therefore, for us to mention, that it is so contrived as to contain about eight inches of the carotid artery of the horse in a vessel filled with water, and made water-tight, except at one point, from which a small horizontal glass tube issues, about an eighth of an inch in diameter. At each contraction of the animal's heart it was found that the water in the small tube advanced two inches and eight-tenths, and that it retired to its former place during the diastole of the heart. The diameter of the artery was seven-twentieths of an inch. Hence it may be calculated that at each pulsation its capacity was increased by about a thirtieth part.

Having ascertained this fact, M. Poiseuille goes on to inquire, whether the power which is expended by the blood in causing this dilatation is restored by the subsequent contraction of the artery.—For this purpose a portion of the common carotid artery of the horse, ten inches long, and seven-twentieths of an inch in diameter, taken immediately after death, was connected with the end *a*, of the tube, (see Figure;) a stop-cock, however, being previously fitted between *a*, and *b*. The other end of the artery was fixed on a tube of analogous construction, different in fact only in so far as the limb *c d* was inclined at about half a right angle instead of being vertical, and the stop cock was placed near the end *d*. The whole of the first tube, the artery and part of the descending limb *b c* of the second tube was filled with water, a little mercury then filled the curvature of the second tube, and the ascending inclined limb of that tube above the mercury was filled with water.—The stop-cock of the last tube being closed, and that on the first tube being opened, mercury was poured into the former by its end *d*, till the pressure on the artery amounted to ninety-five millimetres or about 3.8 inches. The stop-cock of the first tube was then closed, and that on the second tube was opened; upon which the water rose instantaneously in the latter, a portion flowed out at the top, and the remainder then sank a little, and assumed a fixed level. On making the necessary computations, M. Poiseuille found that the point to which the mercury was raised in the second tube at the moment of the contraction of the artery indicated an elevation of one hundred aud ten millimetres or 4.4 inches. Hence *the power with which the arterial coats contract upon themselves after being dilated, exceeds that which is expended in dilating them.* In the present experiment the excess was equivalent to six-tenths of an inch of mercury, or three-nineteenths more than the dilating force. In three other experiments, the excess of the column of mercury was 9-20, 14-20, 19-20 of an inch. When repeated with the artery of an animal which had been killed four days before, the excess was less than 4-20. It is evident, therefore, that whatever force the blood after issuing from the heart loses in consequence of its acting on yielding vessels, is completely restored by the elastic contraction of their parietes.—*Edinburgh Medical and Surgical Journal, July,* 1829, *from the Jour. de Physiologie, Jan.* 1829.—*Am. Journal of Medical Sciences.*

## Figures and Tables

**Figure f1:**